# Supplementary material for: Learning curve and surgical outcome of robotic assisted colorectal surgery with ERAS program
Source: Sci Rep. 2022 Nov 29;12:20566. doi: 10.1038/s41598-022-24665-w (PMC9709162; doi:10.1038/s41598-022-24665-w)
Supplement: Supplementary file 1 — Supplementary Information 1. [file 41598_2022_24665_MOESM1_ESM.zip › LSC Raw data-Ñ[▒K/118/O_ñw╝╨░O▒Kñσ.pdf]

正式摘要(電子簽章：0000002113 75021)

請選擇...reporter

臺中榮民總醫院

Operation Note

[redacted]

- 手術日期：2021/02/25
- 手術醫師：VS 蔣鋒帆，CR 黃偲霖，CR 陳映菁，R 林志安
- Anesthesia : GE

Diagnosis

- Pre-operative diagnosis : Malignant tumor of descending colon
- Post-operative diagnosis : Ditto., s/p laparoscopic left hemicolectomy on 2021/2/25

Surgical Indication

- curative treatment

Operation

- 1. Laparoscopic left hemi-colectomy with end-to-end anastomosis [endo GIA 60-4.1*2 and EEA 28]
  2. Intraoperative colonoscopy : (-)
  3. High ligation of IMA root (+) and IMV. Left colic artery and vein were resected and ligated via endoclip.
  4. Proximal cut end: 8 cm, distal cut end: >15 cm
  5. One 12 Fr. penrose drainage tube was inserted from LLQ of abdomen and inserted into pelvis.
  6. The colon was taken down from the splenic flexure site
  7. Enterolysis
  8. ADHESION BARRIER FILM was placed below the central abdominal wound.

Finding

- 1. A 3x5 cm Bormann type I tumor at DS-colon, with intussuception, the tumor invaded through muscular layer and with serosa retraction, cT3-4N0M0. Tattoo was seen distal to the tumor.
  2. No other gross tumor nor polyps was found
  3. There was no obvious visible liver metastasis, no visible peritoneal seeding
  4. No obvious ascites was noted
  5. Adhesion between S-colon itself.

Procedure

- 1. General endotracheal anesthesia was induced and then the patient was placed in modified Trendelenburg lithotomy position.
  Preoperative antibiotics were given. A Foley catheter and a nasogastric tube were placed. The abdomen was prepped and draped in standard sterile fashion.
  2. A small vertical incision was made right next to the umbilicus and a 12 mm trocar was inserted into the peritoneal cavity by open method. Peumoperitoneum was established with CO2 inflation. Laparoscopy was inserted and then another 12 mm trocar was inserted over RLQ. Extra three 5 mm trochars were inserted over RUQ, LUQ and LLQ of abdomen. The operative findings were mentioned as above. Adhesion lysis was performed via Harmonic scalpel.
  3. The small bowel was inspected and retracted to the right by grasper. The D-colon was then held by grasper and the peritoneum overlying the mesentery was then scored with electrocautery. IMV was identified and ligated. The pancrease area was identified. The left colic artery,vein and IMA root were ligated with endoclips, and transected. Dissection was done above the Gerota's fascia and toward the peritoneal attachments.
  4. The avascular line of Toldt along the left paracolic gutter was incised from the sigmoid colon to

- splenic flexure as possible.
  5. The lesser sac was approached and the omentum was resected carefully. The gastrocolic omentum was divided as far to the left as possible until the splenic flexure was no longer tethered.
  6. Self adhesion of S-colon was lysis, Mesocolon of lower S-colon was clear, and end- GIA 60-4.1 was applied for transection of lower S-colon.
  7. Incision was further extended from the middle umbilicus trochar site. The proximal colon was then taken out for carefully inspection, and to make sure it has an excellent blood supply.
  8. The desired resection site of the distal T-colon was selected, and purstring clamp and Kocher clamp were applied, 1-0 prolene was used for purstring suture, then specimen was transected. EEA 28 was choosed. Anvil was inserted into lumen of T-colon, purstring suture was tied. Then remaining colon was put back into abdominal cavity. Pneumoperitoneum was created again.
  9. Rectum was irrigated with normal saline. End to end anastomosis was completed by EEA 28 circular staple, complete double donut was checked, and air-leak test was done: no air leak.
  9. The possible bleeder was then checked thoroughly.
  10. One 12 Fr. penrose drainage tube was inserted from LLQ of abdomen and inserted into pelvis.
  11. The ADHESION BARRIER FILM was then placed below the central extended wound.
  12. The fascia of midline wound was closed with interrupted sutures of Num. 1 Vicryl. The fascia of the 12 mm
  trochar and other 5 mm trochars were closed with 1-0 Vicryl.
  13. Skin were closed with interrupted 3-0 vicryl, and covered with surgiseal tissue glue.
  14. The patient tolerated the procedure well and was brought to the POR in stable condition.
- Estimated blood loss: minimum
- 器械數量: 正確
- 紗布數量: 正確

Image

- PAINT
  - colon & rectum

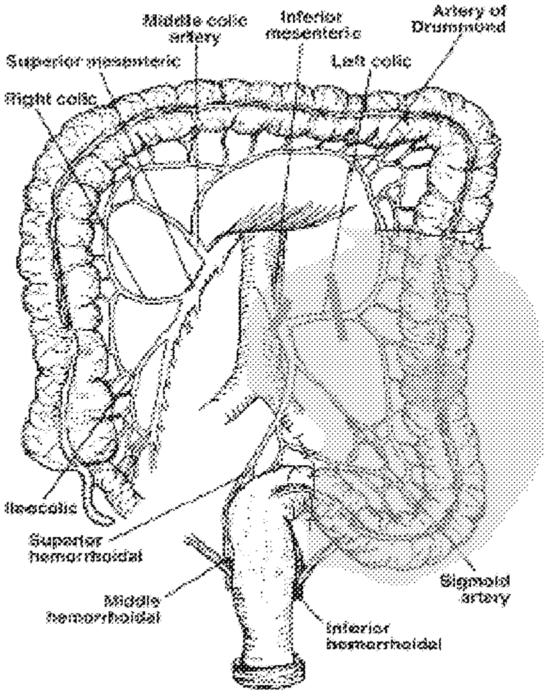

  - abdomen

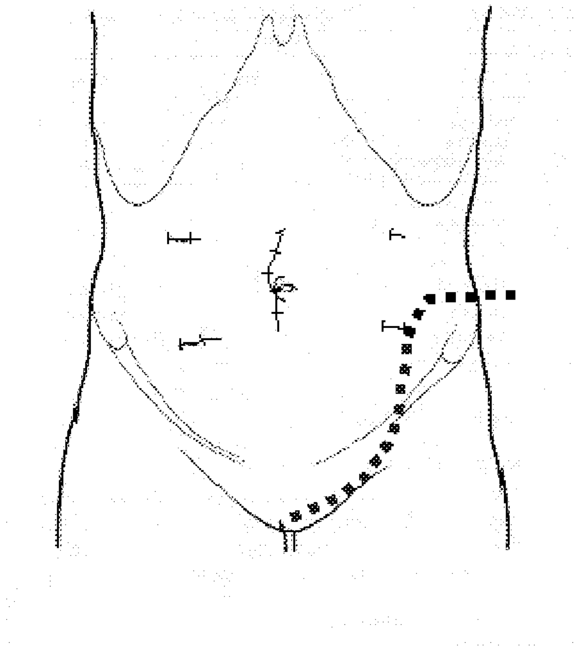

主任 2062F蔣鋒帆 / 醫師 2303K陳映菁

正式摘要(電子簽章：000000211375021)

	請選擇...reporter

doctorRank:B
